# A longitudinal ^18^F-FDG PET/MRI study in asymptomatic stage of genetic Creutzfeldt–Jakob disease linked to G114V mutation

**DOI:** 10.1007/s00415-022-11288-4

**Published:** 2022-07-21

**Authors:** Min Chu, Zhongyun Chen, Binbin Nie, Li Liu, Kexin Xie, Yue Cui, Kewei Chen, Pedro Rosa-Neto, Liyong Wu

**Affiliations:** 1 Department of Neurology, Xuanwu Hospital, Capital Medical University, Changchun Street 45, Beijing, 100053 China; 2 Beijing Engineering Research Center of Radiographic Techniques and Equipment, Institute of High Energy Physics, Chinese Academy of Sciences, Beijing, China; 3 School of Nuclear Science and Technology, University of Chinese Academy of Sciences, Beijing, China; 4 Banner Alzheimer’s Institute, Phoenix, AZ USA; 5 Alzheimer’s Disease Research Unit, McGill Centre for Studies in Aging, Montreal, H4H 1R3 Canada; 6 School of Mathematics and Statistics, Arizona State University, Phoenix, USA

**Keywords:** Genetic Creutzfeldt–Jakob disease, Diffusion-weighted imaging, ^18^F fluorodeoxyglucose-positron emission tomography, Asymptomatic stage

## Abstract

**Background:**

Pathogenic prion protein may start to deposit in some brain regions and cause functional alterations in the asymptomatic stage in Creutzfeldt–Jakob disease. The study aims to determine the trajectory of the brain metabolic changes for prion protein diseases at the preclinical stage.

**Methods:**

At baseline, we enrolled five asymptomatic *PRNP* G114V mutation carriers, six affected genetic *PRNP* E200K CJD patients and 23 normal controls. All participants completed clinical, diffusion-weighted imaging (DWI) and ^18^F fluorodeoxyglucose-positron emission tomography (^18^F-FDG-PET) examinations. Longitudinal follow-up was completed in five asymptomatic mutation carriers. We set three-time points to identify the changing trajectory in the asymptomatic carriers group including baseline, 2-year and 4-year follow-up.

**Results:**

At baseline, DWI signals, the cerebral glucose standardized uptake value rate ratio (SUVR) and clinical status in 5 asymptomatic cases were normal. At the follow-up period, mild hypometabolism on PET images was found in asymptomatic carriers without any DWI abnormal signal. Further group quantitatively analysis showed hypometabolic brain regions in the asymptomatic genetic CJD group were in the insula, frontal, parietal, and temporal lobes in 4-year follow-up. The SUVR changing trajectories of all asymptomatic cases were within the range between the normal controls and affected patients. Notably, the SUVR of one asymptomatic individual whose baseline age was older showed a rapid decline at the last follow-up.

**Conclusions:**

Our study illustrates that the neurodegenerative process associated with genetic CJD may initiate before the clinical presentation of the disease.

## Introduction

Creutzfeldt–Jakob disease (CJD) is a rare, fatal spongiform neurodegenerative disease caused by an accumulation of misfolded prion protein and is characterized by rapidly progressive dementia, motion dysfunction, and akinetic mutism [1]. CJD exists in four clinical pathological forms: sporadic CJD (sCJD), genetic CJD (gCJD), iatrogenic CJD (iCJD), and variant CJD (vCJD) [2]. Approximately 10–15% of prion diseases are genetic variants, carried either by a point mutation or an insertion of octapeptide repeats in the prion protein (*PRNP*) gene [3, 4]. Although the *PRNP* gene is with variable penetrance [5], asymptomatic carriers of PRNP mutation carriers are at high risk of developing CJD which provides an opportunity to characterize the disease trajectory. An intriguing aspect is that although the mutation of *PRNP* is congenital, the clinical affected onset generally occurs at an older age, leaving a time interval free of clinical symptoms and signs. However, the metabolism-changing trajectory of this asymptomatic stage is still not well understood.

The present guidelines for CJD recommend magnetic resonance imaging (MRI), especially diffusion-weighted imaging (DWI) and fluid-attenuated inversion recovery (FLAIR) for sensitive imaging in CJD [1]. Additionally, fluorodeoxyglucose-positron emission tomography (FDG-PET) can detect pathophysiological functional changes in vivo*,* which is confirmed to reflect neuronal and synaptic activity in both asymptomatic and symptomatic stages in prion disease studies [6]. PET is now showing promising significance for the early diagnosis of CJD [7]. In the symptomatic stages of gCJD patients, FDG-PET can detect hypometabolism in the cortical and limbic lobes, basal ganglia, and thalamus [8–11]. In our previous 2-years longitudinal investigation, we found no significant differences in the involvement of brain regions in *PRNP* mutation carriers at an average of 10 years before symptom onset with the use of stringent multiple comparison correction [12]. However, this should be confirmed by further longitudinal follow-up studies.

In this study, we conducted the longitudinal assessment of glucose metabolism using FDG-PET in asymptomatic *PRNP* mutation carriers, to explore their vulnerable brain regions and changing trajectories. All *PRNP* mutation carriers were from the same pedigree which had been reported in our previous work [12–14]. To put our preclinical gCJD research into the context of general gCJD, we also enrolled affected gCJD patients as well as normal controls. We hypothesized that the level of the cerebral metabolism in the gCJD preclinical stage was lower than that in the normal controls but higher than that in affected patients.

## Methods

### Ethics

The study was conducted according to the tenets of the Declaration of Helsinki. The clinical protocols were approved by the ethics committee and local institutional review board of Xuanwu Hospital, Capital Medical University, China. The study was conducted following relevant guidelines and regulations for the use of human subjects in research. Written informed consent was obtained from all participants or their guardians before the start of the study.

### Subjects

Five asymptomatic *PRNP* G114V mutation carriers of a Chinese gCJD family were enrolled in the study. Details of the clinical findings and genetic analysis of this family have already been published [13, 15]. Six genetic CJD patients carried *PRNP* E200K mutations and 23 normal controls were also enrolled from July 1, 2017, to October 31, 2021, in the Department of Neurology at Xuanwu Hospital. All patients were clinically diagnosed as probable CJD cases and carried *PRNP* mutations [1]. Normal controls were age- and sex-matched to the mutation carriers and had no neurological complaints and performed within the normal range on neuropsychological tests (Mini-Mental State Examination [MMSE] score: ≥ 24 and Clinical Dementia Rating [CDR] score: 0).

### Clinical and neuroimaging evaluations

At baseline, all asymptomatic carriers, affected patients, and healthy controls were clinically examined and underwent neurophysiological examination and FDG-PET scan. We measured clinical dementia severity using CDR, with scores ranging from 0 (cognitive normality) to 3 (maximal cognitive impairment), and general cognition function using the MMSE, with scores ranging from 0 (severe impairment) to 30 (no impairment). Only five *PRNP* mutation carriers completed the 4 year longitudinal follow-up and underwent neuropsychological examination and FDG-PET scan every 2 years.

As a routine clinical diagnostic, DWI in axial planes with a slice thickness range of 5 mm was obtained for all subjects. DWI was performed by using a single-shot echo-planar technique (TR 10,000 ms, 1 average, matrix 96 × 128, FOV 26 × 26) with *b* values of 0 and 1000 s/mm2 (TE 101 ms, 1 average). Hyperintensity was visually screened on DWI scans by two independent neuroradiologists (KWC and JZ) with experience in DWI qualitative analysis.

In addition, all subjects received a 2 h electroencephalogram (EEG) at baseline using an 18-lead electroencephalographic transducer (Micromed, Italy). Electrodes were placed following the international standard 10–20 system. Conventional single, double, and sphenoid leads were traced. Periodic sharp wave complexes (PSWCs) are visually screened on the EEG by two independent professional electrophysiological experts (LYW and HY).

### FDG PET/MRI acquisition parameters

All images were acquired on a hybrid 3.0 T TOF PET/MRI scanner (SIGNA PET/MR, GE Healthcare, WI, USA). PET and MRI data were acquired simultaneously using a vendor-supplied 19-channel head and neck union coil. Each subject was asked to fast for at least 6 h to reach a serum level lower than 9 mmol/l and received an intravenous injection of 3.7 MBq/kg ^18^F-FDG. Subjects were asked to wait at least 30 min before FDG administration and during the brain uptake phase. Three-dimensional (3D) T1-weighted sagittal images and ^18^F-FDG PET volumes were acquired during the same session. Static ^18^F-FDG-PET data were acquired in list mode for 30 min and encompass 89 slices covering the whole brain, using the following parameters: matrix size: 192 × 192, FOV: 350 × 350 mm^2^, and pixel size: 1.82 × 1.82 × 2.78 mm^3^, including corrections for random coincidences, dead time, scatter, and photon attenuation. Attenuation correction was performed based on MR imaging of the brain (Atlas-based co-registration of 2-point Dixon). The default attenuation correction sequence was automatically prescribed and acquired as follows: LAVA-Flex (GE Healthcare) axial acquisition, TR: 4 ms, TE: 1.7 ms, slice thickness: 5.2 mm with a 2.6-mm overlap, 120 slices, pixel size: 1.95 × 2.93 mm, and acquisition time: 18 s. The images were reconstructed with a time-of-flight point spread function and the order subset expectation maximization (TOF-PSF-OSEM) algorithm (32 subsets 8 iterations and a 3-mm cut-off filter).

### PET image preprocessing

PET images were preprocessed using SPM12, implemented in MATLAB (MathWorks, Natick, Massachusetts). After spatial normalization of the structural MR images to standard Montreal Neurological Institute (MNI) space using diffeomorphic anatomical registration through exponentiated lie algebra normalization, the transformation parameters determined by the T1-weighted image spatial normalization were applied to the co-registered PET images for PET spatial normalization. The images were then smoothed using an 8-mm full-width half-maximum isotropic Gaussian kernel. Finally, PET scan intensity was normalized using a whole cerebellum reference region to create standardized uptake value ratio (SUVR) images.

### Statistical analysis

PET images statistical analyses were conducted using SPM12, implemented in MATLAB (MathWorks, Natick, Massachusetts). At baseline, the processed ^18^F-FDG PET data were used to perform voxel-wise whole-brain comparisons. Student’s two-tailed *t* test was used to compare asymptomatic mutation carriers, gCJD patients and normal controls, with age and sex as covariates. Comparisons between follow-up and baseline in asymptomatic group were performed using paired *t* tests. The cluster size correction threshold was set at *p* < 0.01 (false discovery rate [FDR]-corrected). Then, to observe the changing tendency trajectory of each asymptomatic subject, we considered the brain regions with a significant difference in the third visit as regions of interest (ROI) and extract the SUVR in these brain regions of asymptomatic cases to delineate a trajectory line chart, by using a scatterplot of SUVR in these brain regions of NC and gCJD as references. Last, we calculated SUVR annual decline rate of each patient using simple linear regression, with SUVR of a given ROI as a dependent variable, and the patient age as an independent variable, considering the gradient as the annual decline rate. If the SUVR annual decline rate of one subject was 2 standard deviations greater than the mean (calculated based on the remaining subjects), we considered this subject as a fast decliner.

Statistical analyses of demographic data were carried out in SPSS 22.0 (IBM Corporation, Armonk, NY, USA). Continuous data are represented as means ± standard deviations. Dichotomous data are represented as absolute values. Group differences were tested using Student’s *t* test for continuous data and Chi-square and Fisher’s exact tests for categorical data. Statistical significance was set at *p* < 0.05.

## Results

### Demographic data and auxiliary examination

The demographic data of the five *G114V* asymptomatic *PRNP* mutation carriers are presented in Table 1. Detailed demographic data and neuropsychological performance in all three groups at baseline are summarized in Table 2. There were no group differences with respect to age (33.80 ± 9.50 vs. 39.00 ± 10.43, *p* = 0.320) and sex (3/2 vs. 10/13, *p* = 0.758) between the asymptomatic *PRNP* mutation carriers and normal controls. The age of affected genetic CJD patients was older than asymptomatic *PRNP* mutation carriers and normal controls. The mean scores of MMSE and CDR were impaired in the affected patients’ group, while were normal in asymptomatic and controls. EEG PSWC patterns were observed in two gCJD patients, but normal in all asymptomatic carriers and controls.

**Table 1:** Demographic data of *G114V* mutation carriers

ID	Sex	Age		
		Baseline	Follow-up 1	Follow-up 2
Subject 1	F	46	48	50
Subject 2	F	42	44	46
Subject 3	F	28	30	32
Subject 4	M	28	30	32
Subject 5	M	25	27	29

*F* female, *M* male

**Table 2:** Demographic data and cognitive status in asymptomatic, affected, and controls groups at baseline

	Asymptomatic (*n* = 5)	Affected (*n* = 6)	Controls (*n* = 23)	*p* value
Age (years)	33.80 ± 9.50	49.00 ± 7.07	39.00 ± 10.43	0.039*
Sex (male/female)	3/2	3/3	10/13	0.788
Disease duration (months)	/	2.31 ± 1.65	/	
MMSE	28.00 ± 1.732	9.00 ± 11.76	27.39 ± 1.20	< 0.001
CDR	0	2.00 ± 1.22	0	< 0.001
DWI hyperintensity (Y/N)	0/5	3/3	0/23	< 0.001
EEG PSWCs (Y/N)	0/5	2/4	0/23	0.007

CDR Clinical Dementia Rating, MMSE Mini-Mental State Examination, PSWCs periodic sharp wave complexes, *Y* yes, *N* no

*Post hoc multiple comparisons: Asymptomatic vs controls, *p* = 0.293; Asymptomatic vs affected, *p* = 0.016; Affected vs controls, *p* = 0.034

**Fig. 1:**
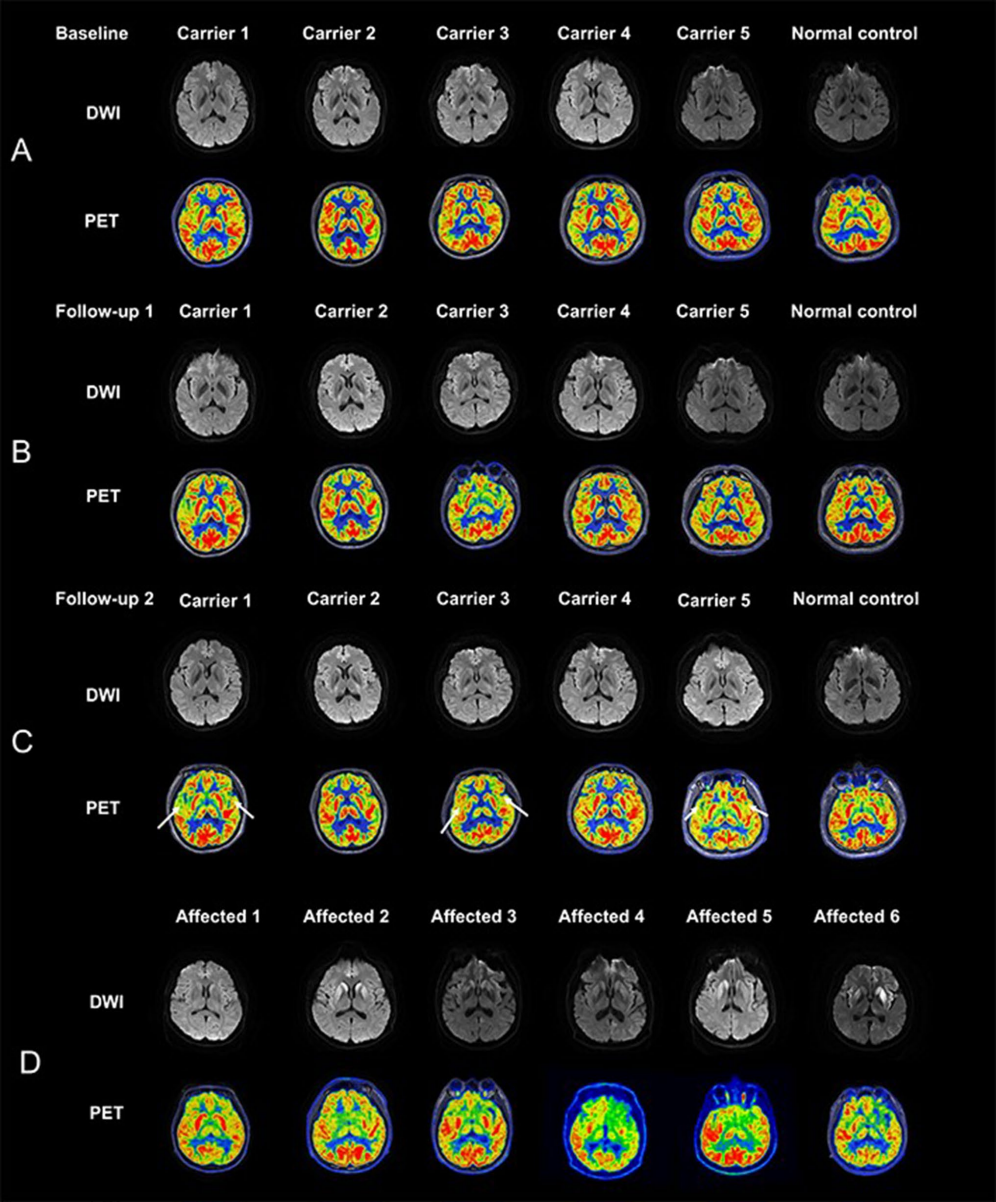
DWI and PET images of asymptomatic PRNP *G114V* carriers and affected gCJD patients. **A** At baseline, DWI and PET images of five asymptomatic *PRNP* carriers and one normal control showed no abnormality. **B** At 2-year follow-up, DWI and PET images of five asymptomatic *PRNP* carriers and one normal control showed no abnormality. **C** At 4-year follow-up, mild hypometabolism was found on PET in carrier 1,3 and 5. No abnormal signal on DWI were found in all subjects (**D**). High signals were found in thalamus, basal ganglia, or cortex on DWI images in affected patients 2, 5 and 6; Hypometabolism in thalamus, basal ganglia or cortex were found in all six affected gCJD patients

The five asymptomatic *PRNP* carriers completed the 4-year follow-up. The DWI and PET images were normal in all asymptomatic carriers at baseline and 2-year follow-up (Baseline and Follow-up 1, Fig. 1A and B). Mild hypometabolism was found on PET images of carriers 1, 3 and 5 at 4-year follow-up, but the DWI images were normal (Follow-up 2, Fig. 1C). Three gCJD patients showed hyperintensity in cortical or basal ganglia on DWI images, and all gCJD patients observed hypometabolism on PET images (Fig. 1D).

### Baseline hypometabolism pattern of asymptomatic and affected gCJD

**Fig. 2:**
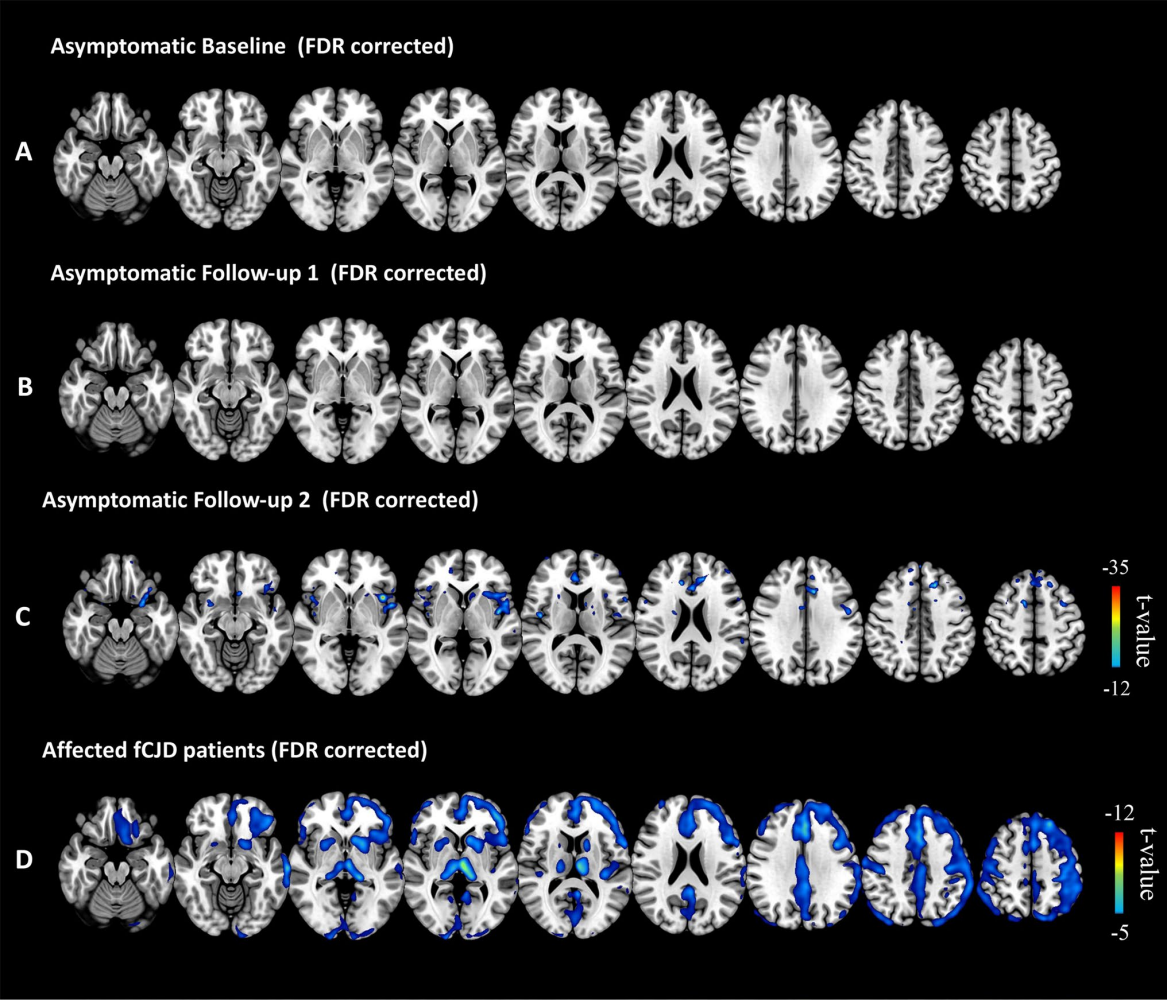
Hypometabolism profile of asymptomatic and affected gCJD patients. **A** No significant voxel survived after FDR-correction in asymptomatic mutation carriers at baseline; **B** no significant voxel survived after FDR-correction in asymptomatic carriers in the first follow-up; **C** regions of hypometabolism in the second follow-up in asymptomatic gCJD group (FDR-correction), mainly distributed in the insula, frontal, parietal, and temporal lobes. The hypometabolism pattern in preclinical stage was within the range of affected gCJD hypometabolism pattern (Fig. 1D); **D** regions of hypometabolism in affected gCJD patients are distributed more diffusedly in the cortical and sub-cortical regions including frontal, temporal, parietal, occipital, insula, basal ganglia, and thalamus (FDR correction)

The baseline hypometabolism pattern of asymptomatic *PRNP* mutation carriers and the symptomatic gCJD group are shown in Fig. 2A and D (FDR correction). At baseline, no significant difference was found between the asymptomatic and normal control groups. Hypometabolisms in the symptomatic gCJD group were distributed more diffusely in the cortical and subcortical regions including the frontal, temporal, parietal, occipital, insula, basal ganglia, and thalamus (FDR correction). Spatial coordinates and peak values of brain areas of hypometabolism in affected gCJD patients are shown in Table 3.

**Table 3:** Spatial coordinates and peak values of brain areas showing significant hypometabolic in asymptomatic at follow-up 2 and affected patients at baseline

	Side	MNI coordinate	Cluster size	*T* value
Asymptomatic (follow-up 2)				
Insula	L	− 40 14 − 2	13,035	− 33.70
Insula	R	44 -8 14	1810	− 23.71
Middle frontal	L	− 42 46 24	226	− 13.71
Superior parietal	R	22 − 42 44	201	− 13.18
Inferior opercular frontal	R	44 4 26	174	− 14.77
Precentral	R	28 − 20 62	162	− 22.66
Superior orbital frontal	L	− 12 58 − 18	106	− 12.91
Superior frontal	R	36 62 14	98	− 14.84
Temporal inferior	L	− 38 10 − 42	56	− 15.37
Superior temporal pole	R	28 22 − 32	56	− 14.29
Affected (baseline)				
Superior frontal	L	− 22 34 52	1246	− 7.96
Thalamus	L	− 4 − 16 6	633	− 10.29
Thalamus	R	16 − 28 2	633	− 7.46
Postcentral	L	− 22 − 38 74	616	− 8.48
Inferior triangular frontal	L	− 46 40 6	509	− 7.97
Temporal pole	R	42 − 28 12	428	− 7.12
Postcentral	R	28 − 36 74	356	− 10.02
Insula	L	− 36 28 − 12	271	− 9.04
Caudate	L	− 12 14 2	238	− 7.56
Putamen	L	− 20 10 − 4	238	− 7.20
Post cingulum	L	− 3 − 47 26	195	− 7.04
Precuneus	L	− 14 − 80 56	129	− 9.21
Inferior parietal	L	− 60 − 42 46	104	− 7.29
Superior orbital frontal	L	− 10 32 − 24	184	− 7.59
Middle occipital	L	− 44 − 74 0	148	− 6.18

MNI Montreal Neurological Institute, *R* right, *L* left

### Follow-up hypometabolism pattern of asymptomatic gCJD

The hypometabolism pattern in the first and second follow-up in asymptomatic *PRNP G114V* mutation carriers are shown in Fig. 2B and C (FDR correction). In the first follow-up, no significant changes were found. In the second follow-up, hypometabolic brain regions were distributed in the insula, frontal, parietal, and temporal lobe (FDR corrected). Spatial coordinates and peak values of brain areas of hypometabolism at second follow-up in asymptomatic gCJD patients are shown in Table 3.

### Trajectory of hypometabolic changes of asymptomatic gCJD

**Fig. 3:**
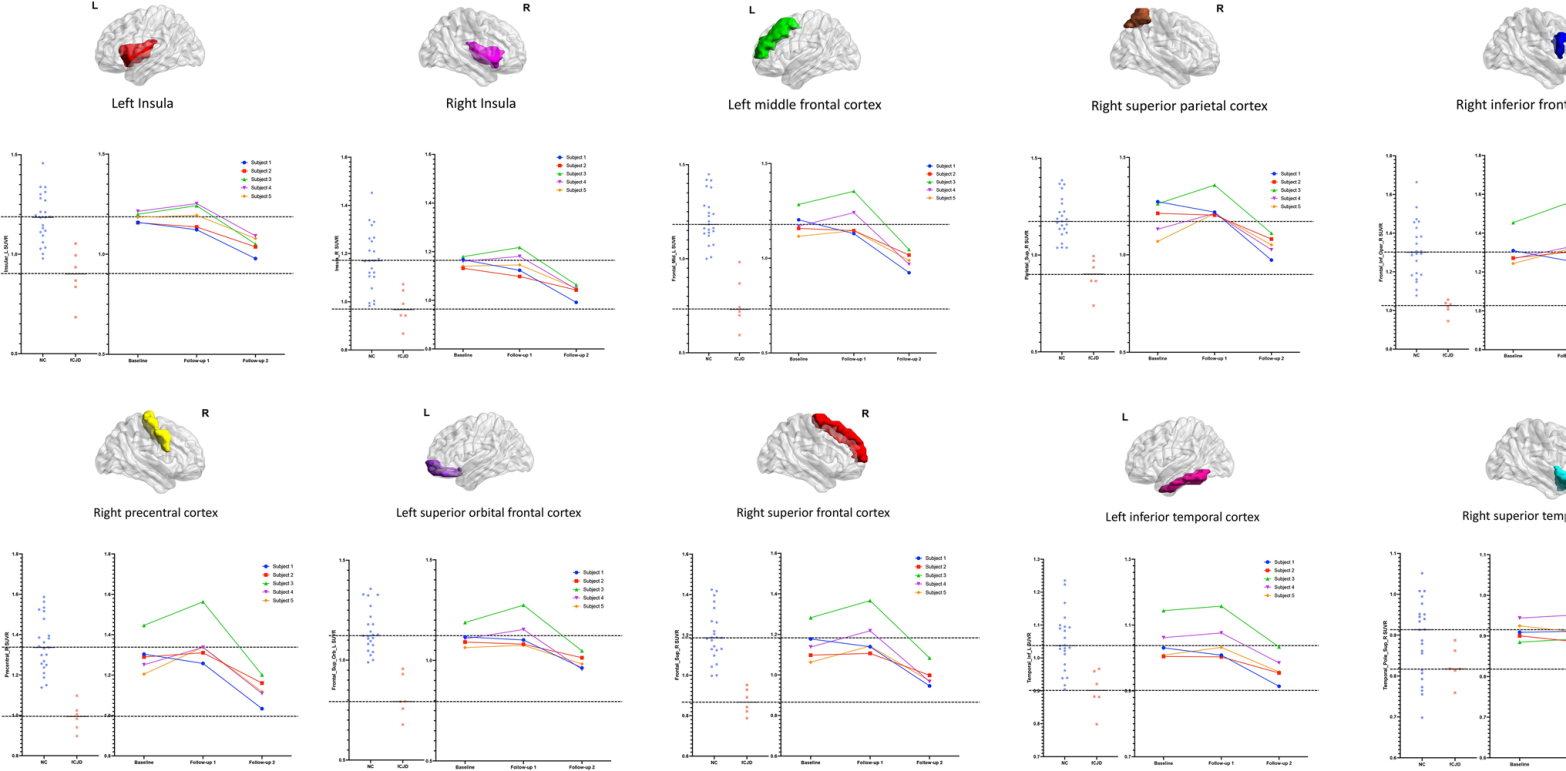
Metabolism changing trajectories of asymptomatic *PRNP G114V* mutation carriers. The dots represent the mean cerebral standardized uptake value ratio (SUVR) of the 10 statistical significantly different brain regions in longitudinal stage, respectively, including bilateral insula, left frontal middle gyrus, right superior parietal gyrus, right inferior frontal operculum, right precentral gyrus, left superior orbital frontal gyrus, right superior frontal gyrus, left inferior temporal gyrus, and right superior temporal pole. The lines at the right side of the chart represent the metabolism changing trajectories in asymptomatic carriers; scatterplot at left side represents the SUVR of these brain regions of normal controls and gCJD patients considered as the reference level

The FDG-PET SUVR of all subjects decreased as the preclinical course progressed, especially in the second follow-up. The trajectories are shown in Fig. 3. The metabolism values of subject 1, who was older at baseline, declined faster and was nearly approaching the mean value of the gCJD disease condition at the third visit. In addition, almost all the metabolism values of the ROI brain region of subject 1 in the follow-up period were below the mean normal range. The annual SUVR decreased rate of subject 1 was greater than the other four subjects and defined as a fast decliner, especially in the temporal, frontal, and limbic brain regions, with a 7.5% reduction in the right superior parietal cortex, 7.0% reduction in left middle frontal cortex, 6.8% reduction in the right precentral regions, 6.3% reduction in the inferior frontal opercular, 5.8% reduction in the right superior frontal cortex, 4.5% reduction in the left insula and 4.4% reduction in the right insula. The detailed metabolic decreased rates of the other four subjects are shown in Table 4.

**Table 4:** Annual percentage decline rate of cerebral metabolism in asymptomatic *PRNP* carriers

		Subject 1 (%)	Subject 2 (%)	Subject 3 (%)	Subject 4 (%)	Subject 5 (%)
Insula	L	− 4.5*	− 3.0	− 3.7	− 3.1	− 2.7
Insula	R	− 4.4*	− 2.2	− 2.9	− 2.8	− 2.3
Middle frontal	L	− 7.0*	− 3.5	− 5.9	− 5.0	− 3.2
Superior parietal	R	− 7.5*	− 3.3	− 3.8	− 2.6	− 0.4
Inferior opercular frontal	R	− 6.3*	− 2.2	− 5.0	− 3.9	− 3.2
Precentral	R	− 6.8*	− 3.2	− 6.1	− 3.6	− 2.2
Superior orbital frontal	L	− 3.9	− 2.0	− 3.5	− 3.8	− 2.0
Superior frontal	R	− 5.8*	− 2.5	− 5.0	− 4.3	− 2.3
Inferior temporal	L	− 2.9	− 1.3	− 2.8	− 1.9	− 1.3

*Represent SUVR of subject 1 was greater than 2 standard deviations above the mean (calculated based on subjects 2, 3, 4 and 5)

## Discussion

In this study, we conducted a longitudinal follow-up by using FDG-PET on asymptomatic PRNP G114V mutation carriers, to explore the characteristics of brain metabolic changes in the preclinical stages of gCJD. Our data indicate that the neurodegenerative process associated with gCJD might begin in the limbic and cortical regions before the clinical presentation of the disease. Our study provides evidence that brain metabolic changes may occur before symptom onset in gCJD and functional deterioration may accelerate when closer to the age of onset.

Hypometabolism was found in the insula, frontal, temporal, and parietal cortices when the PET image was compared between the second follow-up and baseline in five G114V mutation carriers. These voxels of brain regions could survive after FDR correction. So far, to our knowledge, no research in other sites had targeted the disease trajectory of gCJD for more than 4 years in asymptomatic stages. In our previous work [11], we conducted a 2-year follow-up in seven asymptomatic mutation carriers and found no significant brain regions after comparison correction, which was consistent with the present study. In addition, the altered brain regions in asymptomatic stages were reliable and convincing, because all were in the range within those of affected gCJD patients, consistently with the inherent pathophysiological changes of gCJD.

In the second follow-up, all *PRNP* mutation carriers were getting older, especially subject 1, who was the oldest one of these asymptomatic G114V mutation carriers. The annual decline rate in these brain regions were greater than the mean plus 2 standard deviations (mean and standard deviations are calculated in the last four carriers), which suggests the decline rate might be influenced by age. The hypometabolism patterns were distributed mainly in the frontal, temporal, and limbic cortices. The insula is thought to be a crucial hub in many key networks, participating in emotional, cognitive, and motivational processes [16]. The frontal lobe, especially the orbital frontal and precentral regions, is responsible for a multitude of cognitive processes and motor functions [17]. The temporal lobe is also associated with mood state and is functionally closely connected to the frontal lobe and insula [18]. This supports that gCJD patients usually have rapidly cognitive decline and neuropsychiatric symptoms as their initial symptoms.

Normal aging is a confounding factor in the follow-up SUVR calculation. Global cerebral metabolic rate for glucose (CMRglc) has been reported to show a significant decline of approximately 6% per decade with age in previous FDG-PET research [19]. Considering the age effect on the trajectory, we calculated the detailed annual decrease rate of each subject that most of the annual decline rates are greater than > 0.6% in the follow-up timepoint in our study, which confirmed that decline was convincing and not just influenced by aging. The two individuals who were older in our study showed faster hypometabolism tendency closer to the mean disease status but were not affected by neurological dysfunction. Symptoms may appear when some threshold is crossed in the abnormalities of cortical functional changes. Considering the metabolism of two individuals declined rapidly but remained asymptomatic, closer observation and annual neuroimaging follow-up should be performed, as 2-year intervals may be inadequate and unable to capture subtle changes that possibly occur just prior to and at the time of conversion.

The hypometabolism pattern of the affected gCJD group was mainly distributed in the frontal–temporal-parietal cortex, subcortical and cingulate regions, and was more diffuse than the preclinical hypometabolism pattern seen in our study, which was consistent with the previous study in gCJD patients [8–11]. In our *PRNP G114V* gCJD patients, caudate nucleus and putamen displayed high signals on DWI [13], suggesting that subcortical regions are affected in those carrying the *G114V* mutation. However, we did not find any involvement of subcortical structure in our G114V preclinical carriers, indicating that the impairment may start from the cortical region in the early stages and spread into the subcortical regions. This cortical to subcortical spread tendency was also found in affected patients. However, one DWI research revealed the thalamus-striatum impairment may appear in the preclinical stage of gCJD and spread to the cortex [20]. All the preclinical studies were carried out with small sample sizes, different image modalities, and individual discrepancies. This has resulted in inconsistencies in the result interpretation. Further research should be conducted in a more homogenous and larger sample to investigate where the impairment starts and its order of progression in the preclinical stage.

Our study confirmed that the autosomal dominant gCJD in its asymptomatic period might provide a window to explore the earliest stages of the disease process. Evidence had been collected in other neurodegenerative diseases including familial Alzheimer’s disease, Huntington’s disease, frontotemporal dementia, and fatal familial insomnia that some biomarkers can be detected several years before symptom onset, suggesting that the ideal time to start treatment could be before clinical presentation, at a point when a minimal irreversible neuronal loss has occurred, and neurological function is still preserved [6, 21–23]. ^18^F-FDG PET appears to be a sensitive and promising investigation tool in CJD and can be useful to identify CJD from other neurodegeneration disorders [24]. Cerebral hypometabolism was reported more widespread than the histopathologic changes and significantly correlated with the presence of protease-resistant prion protein [25]. Our study offered additional evidence for^18^F-FDG PET utility that it may also be a potential screening tool in the asymptomatic stages of gCJD.

Our study has some limitations. First, the sample was relatively small because of the rarity of gCJD and the challenge to complete the ^18^F-FDG-PET examination in these patients, which affected external generalization. Thus, the conclusion of the present study would be considered a pilot exploratory one that needs further confirmation. Second, the longitudinal follow-up remains insufficient, and no carriers progress to the symptomatic stages, so we cannot capture the dynamic trajectory from asymptomatic to symptomatic stage. Third, we cannot avoid the age effect on glucose hypometabolism; thus, a normal control follow-up parallel to the patients’ group is warranted in our ongoing gCJD longitudinal follow-up research. Last, we selected *PRNP G114V* carriers to represent the asymptomatic group and *E200K* carriers as the affected gCJD patient group, because all the *G114V* patients in the pedigree died without completing the FDG-PET. Some inherent differences may exist between different mutations, which is worthy of further investigation in a relatively larger sample.

## Conclusions

Our study illustrates that the neurodegenerative process associated with gCJD may be involved in the insula, frontal, temporal, and parietal lobes before the clinical presentation of the disease, and the decline rate might be influenced by age. Although there are no effective means to cure this fatal disease, the time interval before clinical disease onset may aid important trials or therapies intended to slow or halt the disease process.

## Data Availability

The datasets used and analyzed during the current study are available from the corresponding author on reasonable request. The data are not publicly available due to privacy or ethical restrictions.

## References

[1] Hermann P, Appleby B, Brandel J (2021). Biomarkers and diagnostic guidelines for sporadic Creutzfeldt–Jakob disease. Lancet Neurol.

[2] Knight R, Will RG (2004). Prion diseases. J Neurol Neurosurg Psychiatry.

[3] Chen C, Dong X (2016). Epidemiological characteristics of human prion diseases. Infect Dis Poverty.

[4] Mastrianni J (2010). The genetics of prion diseases. Genet Med.

[5] Minikel EV, Vallabh SM, Lek M (2016). Quantifying prion disease penetrance using large population control cohorts. Sci Transl Med.

[6] Cortelli P, Perani D, Montagna P (2006). Pre-symptomatic diagnosis in fatal familial insomnia: serial neurophysiological and 18FDG-PET studies. Brain.

[7] Qi C, Zhang JT, Zhao W, Xing XW, Yu SY (2020). Sporadic Creutzfeldt–Jakob disease: a retrospective analysis of 104 cases. Eur Neurol.

[8] Sánchez-Soblechero A, Lozano-Ros A, Gómez-Roldós A, Montoya-Aguirre G, Massot-Tarrús A (2021). E200K familial Creutzfeld–Jakob disease. MRI, EEG, PET and neuropathological correlation in a family. Neurologia (Barcelona, Spain).

[9] Mente K, O'Donnell J, Jones S (2017). Fluorodeoxyglucose positron emission tomography (FDG-PET) correlation of histopathology and MRI in prion disease. Alzheimer Dis Assoc Disord.

[10] Renard D, Castelnovo G, Collombier L, Thouvenot E, Boudousq V (2017). FDG-PET in Creutzfeldt–Jakob disease: analysis of clinical-PET correlation. Prion.

[11] Cistaro A, Cassalia L, Ferrara C (2017). Brain 18F-FDG PET/CT findings in a case of genetic Creutzfeldt–Jakob disease due to V203I heterozygous mutation in the PRNP gene. J Neurol.

[12] Lu H, Jing D, Chen Y (2020). Metabolic changes detected by 18F-FDG PET in the preclinical stage of familial Creutzfeldt–Jakob disease. J Alzheimer's Dis JAD.

[13] Ye J, Han J, Shi Q (2008). Human prion disease with a G114V mutation and epidemiological studies in a Chinese family: a case series. J Med Case Rep.

[14] Jing D, Chen Y, Xie K (2021). White matter integrity involvement in the preclinical stage of familial Creutzfeldt–Jakob disease: a diffusion tensor imaging study. Front Aging Neurosci.

[15] Liu Z, Jia L, Piao Y (2010). Creutzfeldt–Jakob disease with PRNP G114V mutation in a Chinese family. Acta Neurol Scand.

[16] Namkung H, Kim S, Sawa A (2018). The insula: an underestimated brain area in clinical neuroscience, psychiatry, and neurology: (Trends in Neuroscience 40:200–207, 2017). Trends Neurosci.

[17] Chayer C, Freedman M (2001). Frontal lobe functions. Curr Neurol Neurosci Rep.

[18] Keren H, Zheng C, Jangraw D (2021). The temporal representation of experience in subjective mood. Elife.

[19] Petit-Taboué M, Landeau B, Desson J, Desgranges B, Baron J (1998). Effects of healthy aging on the regional cerebral metabolic rate of glucose assessed with statistical parametric mapping. Neuroimage.

[20] Lee H, Rosenmann H, Chapman J (2009). Thalamo-striatal diffusion reductions precede disease onset in prion mutation carriers. Brain.

[21] Bateman R, Xiong C, Benzinger T (2012). Clinical and biomarker changes in dominantly inherited Alzheimer's disease. N Engl J Med.

[22] Tabrizi S, Langbehn D, Leavitt B (2009). Biological and clinical manifestations of Huntington's disease in the longitudinal TRACK-HD study: cross-sectional analysis of baseline data. Lancet Neurol.

[23] Rohrer J, Nicholas J, Cash D (2015). Presymptomatic cognitive and neuroanatomical changes in genetic frontotemporal dementia in the Genetic Frontotemporal dementia Initiative (GENFI) study: a cross-sectional analysis. Lancet Neurol.

[24] Henkel K, Zerr I, Hertel A (2002). Positron emission tomography with [(18)F]FDG in the diagnosis of Creutzfeldt–Jakob disease (CJD). J Neurol.

[25] Cortelli P, Perani D, Parchi P (1997). Cerebral metabolism in fatal familial insomnia: relation to duration, neuropathology, and distribution of protease-resistant prion protein. Neurology.

